# Comparative Analysis of Complete Chloroplast Genomes of *Anemoclema, Anemone, Pulsatilla*, and *Hepatica* Revealing Structural Variations Among Genera in Tribe Anemoneae (Ranunculaceae)

**DOI:** 10.3389/fpls.2018.01097

**Published:** 2018-07-27

**Authors:** Huijie Liu, Jian He, Chuanhua Ding, Rudan Lyu, Linying Pei, Jin Cheng, Lei Xie

**Affiliations:** ^1^School of Nature Conservation, Beijing Forestry University, Beijing, China; ^2^Beijing Forestry University Forest Science Co. Ltd., Beijing, China

**Keywords:** chloroplast genome, inversion, IR expansion, phylogenomics, Ranunculaceae, transposition, Tribe Anemoneae

## Abstract

Structural rearrangements of *Anemone* species' chloroplast genome has been reported based on genetic mapping of restriction sites but has never been confirmed by genomic studies. We used a next-generation sequencing method to characterize the complete chloroplast genomes of five species in the tribe Anemoneae. Plastid genomes were assembled using *de novo* assembling methods combined with conventional Sanger sequencing to fill the gaps. The gene order of the chloroplast genomes of tribe Anemoneae was compared with that of other Ranunculaceae species. Multiple inversions and transpositions were detected in tribe Anemoneae. *Anemoclema, Anemone, Hepatica*, and *Pulsatilla* shared the same gene order, which contained three inversions in the large single copy region (LSC) compared to other Ranunculaceae genera. *Archiclematis, Clematis*, and *Naravelia* shared the same gene order containing two inversions and one transposition in LSC. A roughly 4.4 kb expansion region in inverted repeat (IR) regions was detected in tribe Anemoneae, suggesting that this expansion event may be a synapomorphy for this group. Plastome phylogenomic analyses using parsimony and a Bayesian method with implementation of partitioned models generated a well resolved phylogeny of Ranunculaceae. These results suggest that evaluation of chloroplast genomes may result in improved resolution of family phylogenies. Samples of *Anemone, Hepatica*, and *Pulsatilla* were tested to form paraphyletic grades within tribe Anemoneae. *Anemoclema* was a sister clade to *Clematis*. Structual variation of the plastid genome within tribe Anemoneae provided strong phylogenetic information for Ranunculaceae.

## Introduction

Comparative analysis of chloroplast genomes can provide valuable information for phylogeny reconstruction and resolution of complex evolutionary relationships (Shaw et al., 2007; Mardanov et al., 2008; Moore et al., 2010; Park et al., 2017; Sun et al., 2017). In angiosperms, plastid genome gene number and order is conserved (Wolfe et al., 1987). This is because chloroplast sequences evolve at approximately half the speed of nuclear regions (Jansen et al., 2005; Walker et al., 2014). However, sequence rearrangements in chloroplast genomes have been reported from various kinds of plants (Doyle et al., 1996; Tangphatsornruang et al., 2011; Walker et al., 2015; Sun et al., 2017). These rearrangements include large inversions in large single copy region (LSC), and inverted repeats region (IR) expansions or contractions into single copy regions with inversions (Palmer et al., 1987; Tangphatsornruang et al., 2009). Intramolecular recombination may be the reason for large inversions in plastid genomes (Ogihara et al., 1988; Hiratsuka et al., 1989). These inversion events were probably triggered by tRNA activity (Hiratsuka et al., 1989) or intragenomic recombination in regions with variable G + C content (Fullerton et al., 2001; Smith et al., 2002; Walker et al., 2014). Gene rearrangements and inversions in plastid genomes are believed to have important value in phylogenetic analyses because they are rare, homology estimates are easy, and determination of inversion event polarity is easy (Johansson, 1999; Lee et al., 2007; Jansen et al., 2008; Walker et al., 2014; Yan et al., 2017). Comparison of whole plastid genomes provides the opportunity to explore sequence variation. These comparisons also permit examination of molecular evolutionary patterns associated with structural rearrangement and elucidation of the molecular mechanisms underlying those events.

Ranunculaceae, one of the earliest families that diverged from the eudicots (APG IV, 2016), is composed of more than 2000 mostly herbaceous species with a global distribution (Tamura, 1993, 1995; Ro et al., 1997). In recent years, molecular phylogenetics has provided deep insights and reassessment of Ranunculaceae taxonomy. Some genera have been reduced and a new genus (*Gymnaconitum*) has been proposed based on molecular phylogentic analysis results (Compton and Hedderson, 1997; Compton et al., 1998; Ro et al., 1997; Miikeda et al., 2006; Hoot et al., 2012; Wang et al., 2013; Falck and Lehtonen, 2014; Jiang et al., 2017a). All molecular studies to date were mainly based on tandemly repeated nrDNA and several commonly used plastid regions (Compton and Hedderson, 1997; Compton et al., 1998; Wang et al., 2005, 2010, 2013; Miikeda et al., 2006; Hörandl et al., 2009; Emadzade et al., 2010, 2011; Jabbour and Renner, 2012; Falck and Lehtonen, 2014; Cossard et al., 2016; Jiang et al., 2017a). There are only a few complete chloroplast genomes published and accessible from GenBank (http://www.ncbi.nlm.nih.gov). Phylogenetic inferences for Ranunculaceae taxa based on genomic data have yet to be conducted.

Chloroplast genome structural rearrangements and inversions in *Anemone* and other related genera have been previously reported based on genetic mapping by restriction enzyme site methods (Hoot and Palmer, 1994; Johansson, 1999). In recent years, several complete chloroplast genomes of Ranunculaceae have been published (Chen et al., 2015; Park et al., 2015, 2017; Li et al., 2016; Park and Park, 2016; Jiang et al., 2017b; Lim et al., 2017; Szczecinska et al., 2017; Liu et al., 2018). Recently, Jiang et al. (2017b) and Liu et al. (2018) published plastome sequences of *Anemoclema* and *Clematis s.l*. (including *Archiclematis, Clematis*, and *Naravelia*; Liu et al., 2018), and they also discovered striking structural rearrangements in plastome sequence of the reported genera comparing to that of other Ranunculaceae genera. However, structural variation of tribe Anemoneae plastomes were not discussed in detail by previous studies. The phylogenetic significance of the plastid genome structural variation in tribe Anemoneae and Ranunculaceae still needs to be assessed.

The tribe Anemoneae (as defined by Tamura, 1995) traditionally includes three subtribes (Kingdoniinae, Anemoninae, and Clematidinae), based on previous molecular phylogenetic studies that did not include the subtribe Kingdoniinae (Hoot et al., 2012; Jiang et al., 2017a). For subtribe Clematidinae, almost all satellite genera of *Clematis* (such as *Naravelia* and *Archiclematis*) were nested within *Clematis* by previous studies (Miikeda et al., 2006; Xie et al., 2011; Lehtonen et al., 2016; Jiang et al., 2017a; Liu et al., 2018). In subtribe Anemoninae, Hoot et al. (2012) reduced *Hepatica, Pulsatilla, Oreithales, Knowltonia*, and *Barneoudia* to the genus *Anemone* using molecular phylogenetic results inferred from nrITS and *atpB-rbcL* regions. This result was consistent with subsequent findings by Zhang et al. (2015). However, using six plastid DNA regions, Jiang et al. (2017a) disputed that *Anemone s.l*. (sensu Hoot et al., 2012) was confirmed to be a paraphyletic group and argues that *Hepatica* should not be included in *Anemone s.l*. These two competing phylogenetic hypotheses need to be reconciled using phylogenomic data.

In this study, we report on complete chloroplast genomes from five tribe Anemoneae species (*Anemoclema, Anemone, Pulsatilla*, and *Hepatica*). The plastomes of *Anemone* and *Hepatica* are reported for the first time. Together with the plastome sequence analyses of *Clematis s.l*., the aims of this study are to present whole chloroplast genome data for these species; to compare the plastid genomic structure and sequence variation within the tribe Anemoneae; to test two alternative hypotheses (tRNA activity or G + C content variation) that may cause gene rearrangement events; to test competing phylogenetic hypotheses within tribe Anemoneae using plastid phylogenomic data; and to clarify the phylogenetic significance of plastome structural variation of tribe Anemoneae. We also identified repeat sequences and SSRs inside these five plastomes. The data presented in this study should be useful for future phylogenetic studies of tribe Anemoneae and possibly the rest of the buttercup family.

## Materials and methods

### Plant sampling

We chose to sample five species, *Anemone tomentosa, A. trullifolia, Hepatica henryi, Pulsatilla chinensis*, and *Anemoclema glaucifolium*, for this study. Fresh young plant leaves were collected from the field for DNA extraction and were dried with silica-gel. Vouchers were deposited at the Beijing Forestry University (BJFC) Herbarium. In our previous study (Liu et al., 2018), complete chloroplast genome sequences of *Clematis, Archiclematis*, and *Naravelia* were reported. Thus, in this study, we included samples representing all of the major clades (*Hepatica* clade, *Anemone s.l*. clade, *Anemoclema*, and *Clematis s.l*. clade) of tribe Anemoneae (Hoot et al., 2012; Zhang et al., 2015; Jiang et al., 2017a) to check plastome structural variation. We also obtained whole chloroplast genomes of other Ranunculaceae species and outgroup species of *Berberis* available from Genbank for structural comparison and phylogenomic analysis (Table 1).

**Table 1:** Reference information for sequenced chloroplast genomes.

**Species**	**Sample locality**	**Voucher (Herbarium)**	**Genbank accession**	**Reference**
*Aconitum chiisanense*	Incheon, Korea	VP0000494117 (NIBR)	KT820665	Lim et al., 2017
*Anemone tomentosa*	Barkam, Sichuan, China	*H. J. Liu* I”-1080 (BJFC)	MG001339	This study
*Anemone trullifolia*	Dinggye, Xizang, China	*PE2013* Tibet 2588 (PE)	MH205608	This study
*Anemoclema glaucifolium*	Shangrila, Yunnan, China	*B.Xu-*M417-090(SWFC)	MH205609	This study
*Archiclematis alternata*	Nyalam, Xizang, China	*PE2010 Tibet* 963 (PE)	MG675221	Liu et al., 2018
*Berberis amurensis*	NA	NA	KM057374	Unpublished
*Clematis brevicaudata*	Jiufeng, Beijing, China	*L. Xie* 20140706 (BJFC)	MG675223	Liu et al., 2018
*Clematis fusca* var. *coreana*	NA	NA	KM652489	Park and Park, 2016
*Clematis repens*	Emei, Sichuan, China	*L. Xie 2015*EM24 (BJFC)	MG675222	Liu et al., 2018
*Clematis terniflora*	Huzhou, Zhejiang Prov. China	Unknown number (HZU)	KJ956785	Li et al., 2016
*Coptis chinensis*	NA	NA	KY120323	Unpublished
*Gymnaconitum gymnandrum*	NA	NA	KT964697	Unpublished
Hepatica henryi*	Emei, Sichuan, China	*L. Xie* 2015EM039 (BJFC)	MG001340	This study
*Hydrastis canadensis*	NA	NA	KY085918	Unpublished
*Megaleranthis saniculifolia*	Mt. Sobaek, Korea	Unknown number (Korea University Herbarium)	FJ597983	Kim et al., 2009
*Naravelia pilulifera*	Yangchun, Guangdong, China	*L. Xie & S. Liao* 2014022 (BJFC)	KY120887	Liu et al., 2018
*Naravelia zeylanica*	Machanbaw, Kachin State, Myanmar	*PT-ET* 1281 (PE)	MG675224	Liu et al., 2018
Pulsatilla chinensis*	Yanqing, Beijing, China	*L.Xie* 2015YQ002 (BJFC)	MG001341	This study
*Pulsatilla vernalis*	NA	NA	KR297062	Unpublished
*Ranunculus macranthus*	NA	NA	DQ359689	Raubeson et al., 2007
*Thalictrum coreanum*	Gangwon-do, Korea	NA	KM206568	Park et al., 2015
*Trollius chinensis*	NA	NA	KX752098	Unpublished

*Species with asterisks were collected by this study, whereas others were obtained from Genbank. NA, not applicable*.

### Sequencing, plastome assembly, annotation, and visualization

Approximately 50 mg dried leaves were ground for each species, and total DNAs were extracted using cetyl-trimethylammonium bromide (CTAB; Doyle and Doyle, 1987) with the quality of DNAs assessed by agarose gel electrophoresis. Total DNAs were subsequently sent to Novogene (http://www.novogene.com, China) for short insert (350 bp) library construction and next-generation sequencing. Pair end reads of 2 × 150 bp for all tested species were generated on an Illumina Hiseq 4000 genome analyzer platform. Original reads were filtered using the FASTX-Toolkit (http://hannonlab.cshl.edu/fastx_toolkit) to acquire high-quality data by deleting adaptors and low quality reads.

We used BLAT analysis with a Python script (Weitemier et al., 2014) to exclude nuclear and mitochondrial reads using published plastomes from Ranunculaceae as references. Putative chloroplast reads were used for *de novo* assembly to reconstruct the samples' complete plastid genomes using Geneious R11 (Kearse et al., 2012) with a low sensitivity setting. Contigs from *de novo* assembly were annotated in Geneious R11 and then were concatenated into larger contigs based on *Pulsatilla vernalis* (KR297062) plastomes. All gaps were bridged using conventional Sanger sequencing. The IR region was determined using the Repeat Finder function in Geneious R11. The IR region was subsequently manually inverted and copied to construct the complete plastome sequence. The IR and SC boundaries for all three species were checked using Sanger sequencing.

Complete plastid genomes were manually edited to remove ambiguous sites. The three plastomes were then annotated using the Unix program Plann (Huang and Cronk, 2015) and the annotations were verified using the online program DOGMA (http://dogma.ccbb.utexas.edu/; Wyman et al., 2004). If ambiguous annotations were present between Plann and DOGMA, we determined gene boundaries using the online program Blast (Gish and States, 1993). Illustrations of circular plastoms were generated using the Organellar Genome DRAW tool (Lohse et al., 2013). Final plastid genomes were deposited in GenBank (Table 1).

### Genome comparisions

We obtained other complete chloroplast genomes of Ranunculaceae and an outgroup species *Berberis* from Genbank (Table 1) for comparative analyses. The IR/SC boundary regions of species from tribe Anemoneae were illustrated and compared to other Ranunculaceae species and *Berberis*. MAFFT was used to compare the similarity of plastid genome sequences (Katoh et al., 2005), and mVISTA was used to export visual results to evaluate similarity (Frazer et al., 2004). Visual results from mVISTA were further analyzed using two alignment programs: LAGAN, which produces true multiple alignments regardless of whether they contain inversions or not, and Shuffle-LAGAN, which can detect rearrangements and inversions (Brudno et al., 2003a,b). Detailed gene inversions and transpositions were identified by comparing the gene order of Ranunculaceae samples to *Berberis* with a whole plastome alignment method that used Mauve v2.3.1. (Darling et al., 2010).

### Analysis of G + C content at inversion/transposition borders and sliding window analysis

The G + C content was calculated for the spacer regions boundary each of the major inversions and for transpositions of the tribe Anemoneae' plastomes. Flanking regions were defined as the noncoding sequence between the nearest coding genes on either side of the inversion/transposition boundary (Walker et al., 2014). We also conducted a sliding window analysis to identify the nucleotide variability (Pi) in the inversion/transposition regions using DnaSP version 5 (Librado and Rozas, 2009).

### Characterization of repeat sequences and SSRs

We used REPuter (Kurtz et al., 2001) to identify and locate the repeat sequences for the newly sampled species, including direct, reverse and palindromic repeats, within the plastid genome. For repeat identification, the following parameters were used: (1) 30 bp minimum repeat size and (2) 90% or greater sequence identity (Hamming distance = 3). In order to avoid redundancy, repeat sequence analysis was carried out with a single IR region. SSRs were determined using MISA (Thiel et al., 2003) and parameters were set to 10 repeat units ≥10 for mononucleotide SSRs, six repeat units ≥6 for dinucleotide, five repeat units ≥5 for trinucleotide, four repeat units ≥4 for tetranucleotide, and three repeat units ≥3 for pentanucleotide and hexanucleotide SSRs.

### Phylogenomic analysis

We used the plastome sequences of all the other genera of Ranunculaceae available from GenBank for phylogenomic analysis. The sampling covered 15 of the family's genera, seven of which belong to tribe Anemoneae. There is an accession of *Actaea* plastome (NC034704, unpublished) sequence available in GenBank. However, after extracting genes from this plastome and doing Blast in GenBank, we found that this sample belongs to Apiaceae and was mis-identified as *Actaea*. Thus, we did not include this sequence in the present analysis. *Berberis amurensis* plastome was chosen as the outgroup (Table 1).

In this study, we first separated the complete plastome sequences into coding regions (protein-coding genes, as well as tRNA genes and rRNA genes), intergenic spacer regions, and introns. Gene orders for all samples were tested using mVISTA, and then were shuffled in the same order with a *Berberis amurensis* plastome sequence whenever gene inversions/transpositions were found. All data sets were then aligned using MAFFT v6.833 (Katoh et al., 2005) and manually adjusted by MEGA 7.0 (Kumar et al., 2016). The ambiguous alignments were removed from the data sets for phylogeny reconstruction. Substitution models and data partitions for Bayesian analysis was determined by PartitionFinder v2.1.1 (Lanfear et al., 2016) and the best scheme selected by Akaike information criterion (AIC; Posada and Buckley, 2004).

Phylogeny reconstruction occurred using data sets of LSC, SSC, IR, and complete plastomes with Parsimony (MP) and Bayesian methods. Each data set was further separated into CDs, intron, intergenic spacer regions (Ma et al., 2014).

Parsimony (MP) analysis was conducted for all the separated data sets and the complete plastome data set using PAUP^*^ 4.0b10 (Swofford, 2003). All characters were treated as unordered and equally weighted, and gaps were set as missing data. We used Branch-and-Bound or heuristic search (1000 replicates), simple addition, and tree bisection-reconnection branch swapping with MUL-trees to search the MP tree(s). Branch support values were assessed by performing 1000 bootstrap replicates using 1000 random taxon addition replicates with 10 trees held at each step and TBR swapping.

Bayesian inference (BI) was conducted with MrBayes v3.2.3 (Ronquist and Huelsenbeck, 2003) using partitioned substitution models tested by PartitionFinder. Two independent Markov chain Monte Carlo (MCMC) chains were run, each with three heated and one cold chain for 2,000,000 generations and sampling trees every 100 generations. The MCMC convergence in Bayesian inference was checked by AWTY (https://github.com/danlwarren/RWTY, Warren et al., 2017). The first 20% of trees were discarded as burn-in with the remaining trees being used for generating the consensus tree.

## Results

### Strategies for assembling plastomes

We obtained 2.7 Gb of average NGS clean data for each species, with minimums and maximums of 2.2 Gb (*Pulsatila chinensis*, 6,309,984 reads), and 3.3 Gb (*Anemone tomentosa*, 9,512,414 reads), respectively. Blat analysis selected out 542,906 putative plastid reads for *Anemone tomentosa*, 451,685 reads for *A. trullifolia*, 100,695 reads for *Pulsatilla*, 128,187 reads for *Hepatica*, and 335,675 reads for *Anemoclema*. *Anemone tomentosa* reads obtained three large contigs from *de novo* assembly (83,809 bp, 27,444 bp, and 20,496 bp). Three gaps were bridged using sanger sequencing. For *A. trullifolia* data, only one large contig (126,074 bp) was derived that included complete LSC, IR, and SSC regions and regions without gaps. For *Pulsatilla chinensis* plastid reads, eight large contigs ranging from 3,287 bp to 38,885 bp were obtained. We filled seven gaps for this sample. For *Hepatica henryi*, two large contigs (45,926 bp and 65,671 bp) were obtained and one large gap (ca. 1.5 kb) was filled by designing two pairs of primers. For *Anemoclema* data, three large contigs (92,619 bp, 33,225 bp, and 4,489 bp.) were concatenated. Two gaps were bridged using Sanger sequencing. Information for all of the gaps and primers for Sanger sequencing are presented in the Supplementary Materials.

### Size and structure of plastomes of the samples

**Figure 1:**
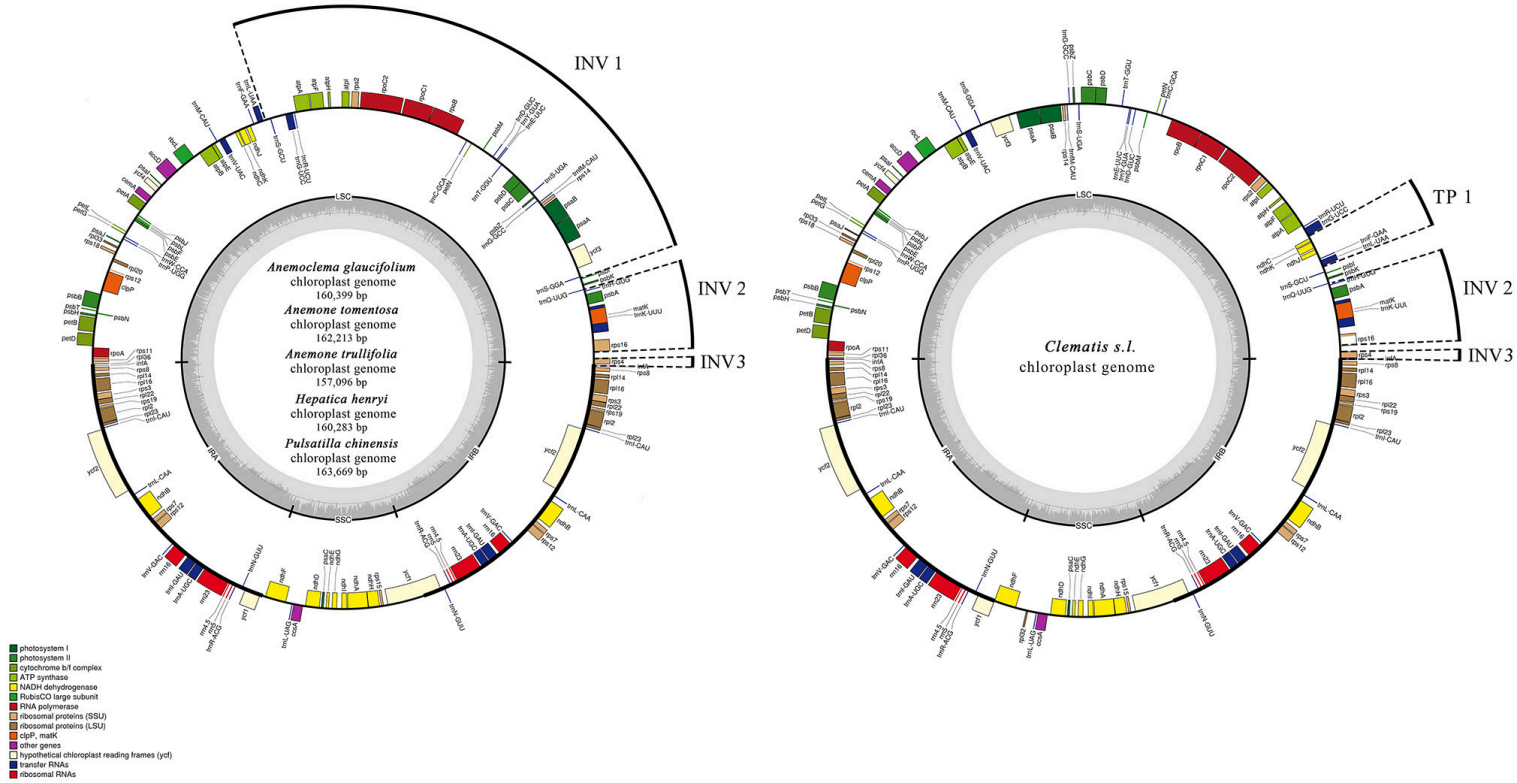
Chloroplast genome maps for *Anemoclema, Anemone, Hepatica*, and *Pulsatilla* samples (lefe), and *Clematis s.l*. (right). Thick lines on the outer complete circle identify the inverted repeat regions (IRa and IRb). The innermost track of the plastome shows the G + C content. Genes on the outside of the map are transcribed in a clockwise direction and genes on the inside of the map are transcribed in a counter clockwise direction. INV, inversion; TP, transposition; IR, inverted repeats; LSC, large single copy; SSC, small single copy; Pi, nucleotide variability.

The genome size of the five newly sequenced samples ranged from 157,096 bp (*Anemone trullifolia*) to 163,669 bp (*Pulsatilla chinensis*; Figure 1), and the overall GC content varied from 37.2% (*Pulsatilla chinensis*) to 37.9% (*Anemoclema glaucifolium* and *Hepatica henryi*; Table 2). All five plastid genomes consisted of a pair of IRs (31,022–31,490 bp) separated by the LSC (78,795–82,339 bp) and SSC (16,257–19,100 bp) regions, respectively (Table 2). The plastomes of the five samples encoded an identical set of 112 genes, including 78 protein-coding genes, 29 transfer RNAs, four ribosomal RNAs, and 25 genes are duplicated in IRs. There are 18 genes with introns in the plastomes of *Anemoclema, Anemone tomentosa*, and *Pulsatilla chinensis*, whereas, 17 genes with introns are present in *Anemone trullifolia* and *Hepatica henryi* plastomes (no introns in *rps16*).

**Table 2:** Base compositions of five newly sequenced Anemoneae' plastomes.

**Features**	***Anemone tomentosa***	***Anemone trullifolia***	***Anemoclema glaucifolium***	***Pulsatilla chinensis***	***Hepatica henryi***
Genome size	162,213	157096	160399	163,669	160,283
Length of LSC	82,327	78795	80250	82,339	80,558
Length of SSC	16,906	16257	17637	19,100	17,647
Length of IR	31,490	31022	31256	31,115	31,039
Total G + C content (%)	37.6%	37.6%	37.9	37.2%	37.9%
Total number of genes	112	112	112	112	112
Protein encoding	79	79	79	79	79
tRNA	29	29	29	29	29
rRNA	4	4	4	4	4
Genes with introns	18	17	18	18	17
Duplicated in IRs	25	25	25	25	25

### Chloroplast genome comparison

**Figure 2:**
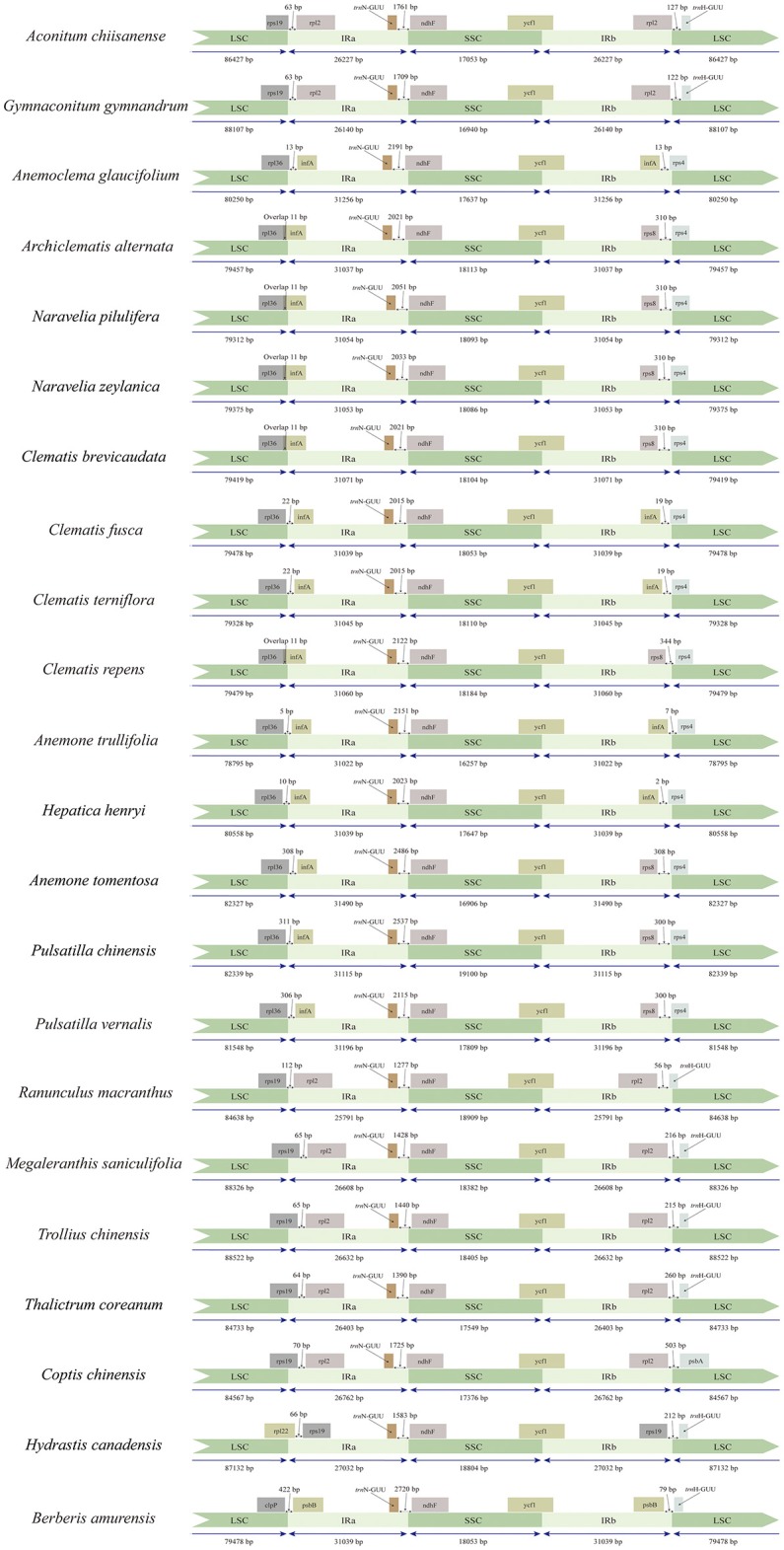
Comparison of the LSC, IRs and SSC boundary regions of tribe Anemoneae, published Ranunculaceae genera, and *Berberis* plastomes.

We compared the IR/SC boundary regions of tribe Anemoneae to other Ranunculaceae species as well as an outgroup from *Berberis*. The junction positions are similar and conserved in tribe Anemoneae, yet differ from other Ranunculaceae species and *Berberis* (Figure 2). For example, *rps19-infA* (seven genes) is in the LSC region of *Berberis, Hydrastis, Coptis, Thalictrum, Megaleranthis, Ranunculus, Aconitum*, and *Trollius* plastomes, but is located in IR in *Clematis* (including *Archiclematis* and *Naravelia*), *Anemoclema, Anomone, Pulsatilla*, and *Hepatica*). This difference makes the IR region of tribe Anemoneae roughly 4.4 kb longer than in other genera of the family. The gene orders located within the IR-SSC and IR-LSC boundaries are similar among tribe Anemoneae samples but different from those in other Ranunculaceae genera (Figure 1).

**Figure 3:**
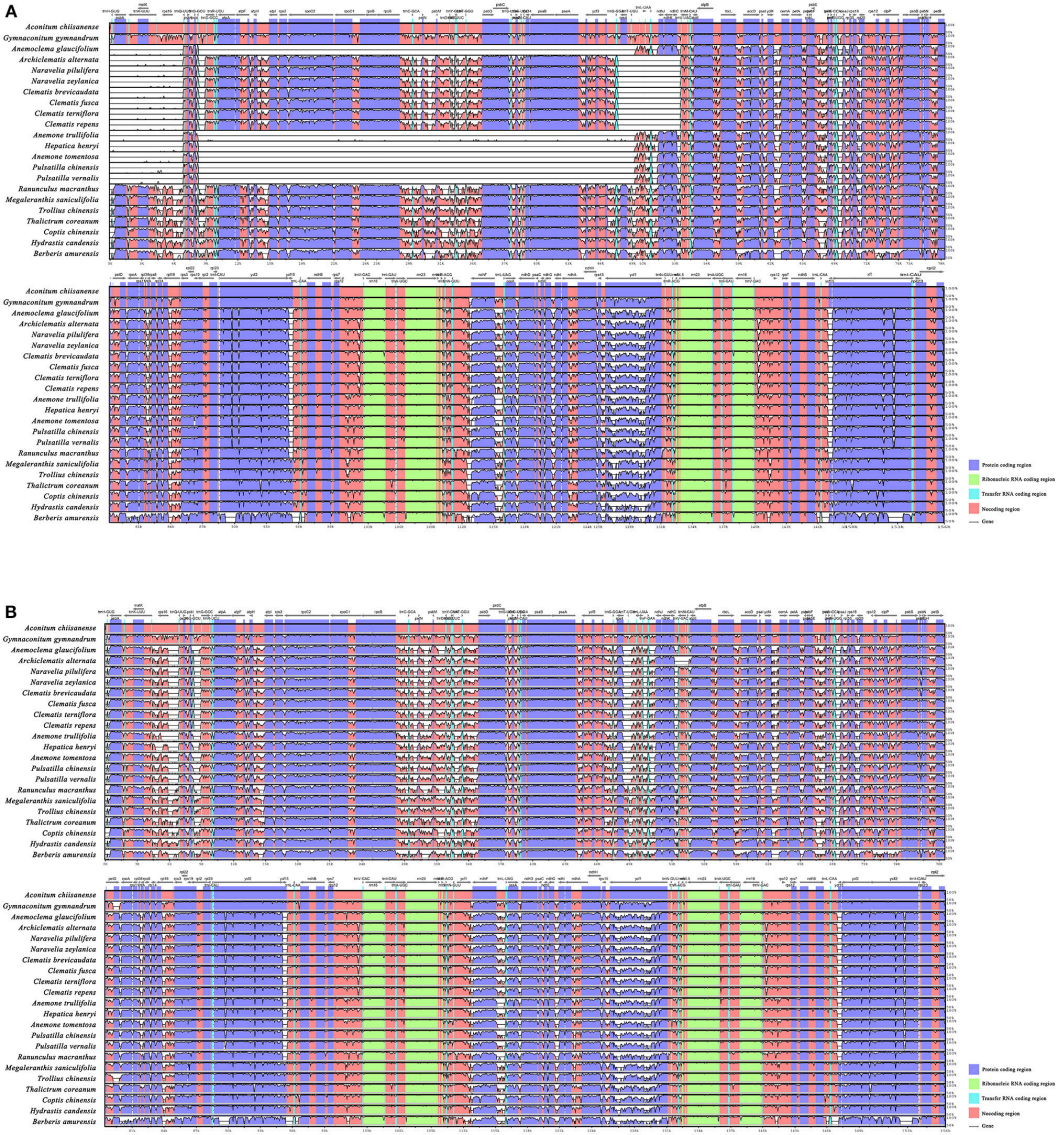
Sequence alignment of tribe Anemoneae, published Ranunculaceae genera, and *Berberis* plastome using the mVISTA program. A cut-off of 70% similarity was used for the plot and the Y-scale represents the percent similarity ranging from 50-100%. Blue represents coding regions, and pink represents non-coding regions. **(A)** LAGAN method (as described in Materials and Methods); **(B)** Shuffle LAGAN method (as described in Materials and Methods).

To investigate levels of genome divergence, multiple alignments of plastid genomes were performed (Figures 3A,B and Supplementary Materials). For mVISTA analysis, LAGAN, and Shuffle-LAGAN program results differed because of gene inversion/transposition occurring in tribe Anemoneae (Figures 3A,B). When using the LAGAN method (Figure 3A), plastomes of *Anemoclema, Anemone, Pulsatilla*, and *Hepatica* showed consistency in gene order, but carried large unalignable regions in LSC comparing to *Clematis* and other Ranunculaceae species. Plastomes of *Clematis s.l*. also showed some unalignable regions compared to other Ranunculaceae species. Conversely, all of the sequences aligned well when Shuffle-LAGAN methods were used (Figure 3B). This approach also revealed high sequence similarity across the coding region accompanied by more variability in non-coding regions.

tribe Anemoneae samples exhibited inversion and transposition regions that were detected by MAUVE (Supplementary Materials), compared to other Ranunculaceae plastomes. In *Anemoclema, Anemone, Hepatica*, and *Pulsatilla*, three inversions are present in the LSC region (Figure 1). The first inversion 1 (INV 1) is located between *trnS-GGA* and *trnS-GCU* (ca. 40 kb in length). The second inversion (INV 2) was between *rps16* and *trnH-GUG* (ca. 5k in length). The third inversion region (INV3) was located in *rps4* (ca. 600 bp in length). Two inversions and one transposition were detected in *Clematis s.l*. plastomes, with INV2, and INV3 being similar to those in *Anemone s. l*. plastomes. The transposition region 1 (TP1) was located between *trnL UAA* and *ndhC* (ca. 3k in length; Figure 1). No inversions or transpositions were found in published plastomes of other Ranunculaceae genera or in comparison to other plastomes of angiosperm species like *Amborella trichopoda, Berberis amurensis*, and *Nicotiana tabacum*.

### G + C content at inversion and transposition borders and sliding window analysis

**Figure 4:**
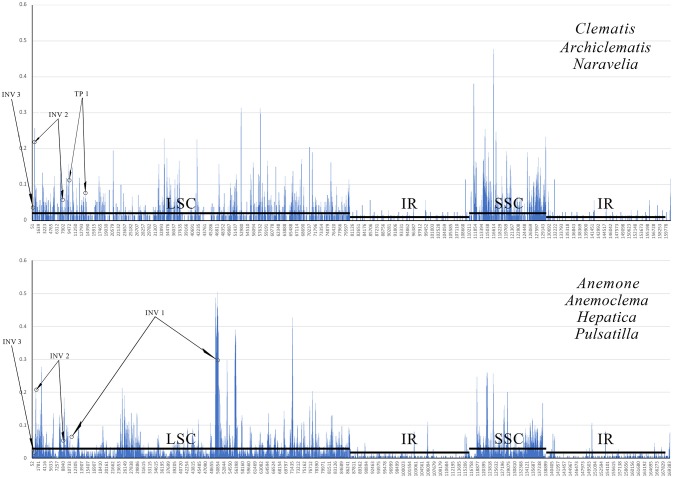
A sliding window analyses of the whole plastid genomes of *Clematis s.l*. (upper) and *Anemoclema, Anemone, Hepatica*, and *Pulsatilla* samples (lower). Circles identify the regions bordering inversion sites, and lines parallel to the x-axis identify the positions of the LSC, SSC and IR regions.

The G + C content of the sequence in boundary regions was detected to be lower than the average G + C content of whole plastome and all the noncoding regions (Table 3). The sliding window analysis revealed that higher nucleotide variability (Pi) was exhibited at SC regions in comparison to IR regions (Figure 4). Genetic variation was particularly high at the boundary of INV1, which existed in *Anemoclema, Anemone, Hepatica*, and *Pulsatilla* plastomes. The INV2 and TP1, found in *Clematis s.l*. plastomes, has a high nucleotide varability at the borders as well. However, we did not find high genetic variation in INV3, which is shared among all the Anemoneae species.

**Table 3:** Percent of G + C content of the whole plastomes, noncoding regions and the regions boundary INV 1, INV 2, INV3, and TP1 in Anemoneae samples.

**Species**	**Whole plastome**	**Noncoding**	**Inversion 1**		**Inversion 2**		**Inversion 3**		**Transposition 1**	
			***psb*I—*trn*S-GGA**	***trn*S-GCU—*trn*L-UAA**	***trn*Q-UUG—*trn*H-GUG**	***rps*16—*rps*4**	***rps*16—*rps*4**	***rps*4—*rps*8**	***trn*G-UCC—*ndh*C**	***trn*L-UAA—*trn*S-GCU**
*Archiclematis alternata*	38.00%	34.60%	NA	NA	23.70%	28.90%	28.90%	33.20%	23.50%	28.00%
*Clematis brevicaudata*	38.00%	34.60%	NA	NA	24.80%	29.20%	29.20%	34.10%	22.30%	28.10%
*Clematis fusca* var. *coreana*	38.00%	34.60%	NA	NA	25.00%	29.10%	29.10%	33.70%	23.20%	28.00%
*Clematis repens*	38.00%	34.60%	NA	NA	25.00%	29.60%	29.60%	34.00%	23.70%	28.00%
*Clematis terniflora*	38.00%	34.60%	NA	NA	24.60%	29.60%	29.60%	33.50%	23.20%	28.20%
*Naravelia pilulifera*	37.90%	34.50%	NA	NA	25.20%	29.00%	29.00%	32.60%	23.00%	27.30%
*Naravelia zeylanica*	37.90%	34.50%	NA	NA	25.80%	29.10%	29.10%	32.90%	22.50%	27.10%
*Anemoclema glaucifolium*	37.90%	34.40%	25.30%	27.90%	28.90%	29.20%	29.20%	33.10%	NA	NA
*Anemone tomentosa*	37.60%	33.80%	25.70%	26.40%	26.20%	25.50%	25.50%	33.40%	NA	NA
*Anemone trullifolia*	37.60%	33.90%	21.30%	26.50%	26.40%	26.60%	26.60%	32.80%	NA	NA
*Pulsatilla chinensis*	37.20%	33.00%	22.80%	27.20%	26.20%	28.00%	28.00%	33.30%	NA	NA
*Hepatica henryi*	37.90%	34.40%	21.70%	27.60%	29.50%	28.90%	28.90%	33.50%	NA	NA
Mean	37.83%	34.29%	23.36%	27.12%	25.94%	28.56%	28.56%	33.34%	26.34%	27.81%

*NA, not applicable*.

### Repetitive sequences

**Figure 5:**
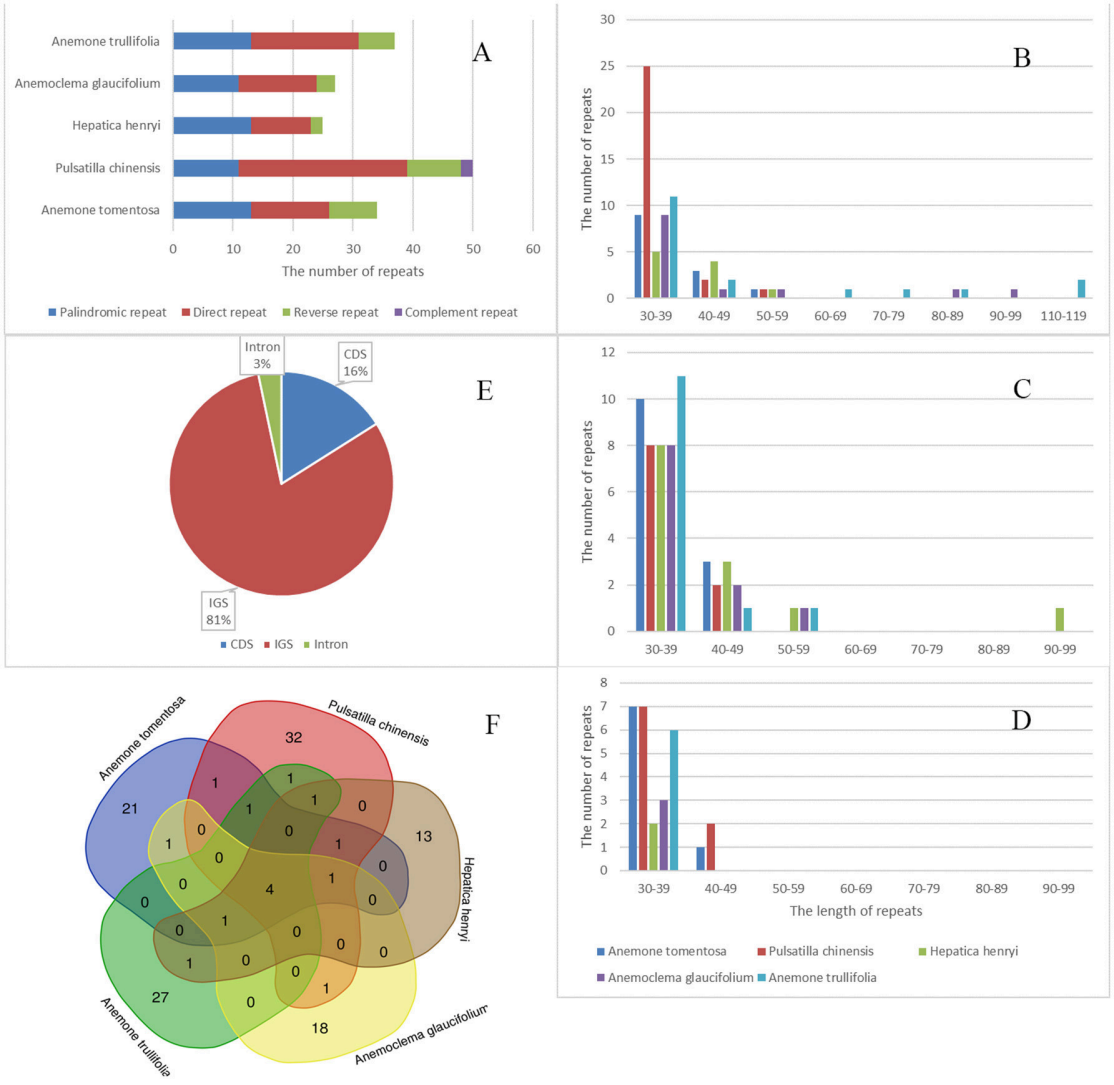
Analyses of repeated sequences in five newly sequenced plastomes. **(A)** number of four repeat types; **(B)** Frequency of direct repeats by length; **(C)** Frequency of reverse repeats by length; **(D)** Frequency of palindromic repeats by length; **(E)** Location of repeats; **(F)** Summary of shared repeats among the five plastomes.

We detected a total of 173 repeats including direct, reverse, palindromic and complement repeats in the five newly sequenced plastomes (Figure 5). The most common repeat types are direct repeats, which account for 45% of the total repeats, followed by palindromic repeats (35%) and reverse repeats (16%). The only two complement repeats were found in *Pulsatilla* plastomes. Most of the repeats were short, ranging from 30–59 bp. However, a few much longer direct and reverse repeats (up to more than100 bp) were found in *Hepatica* and *Anemone* plastomes. The majority of repeats were located in noncoding regions (84%), among which only 3% were found in introns. There were 16% repeats detected in CDs.

We also investigated repeats that are shared among plastomes of the five samples by using strict criteria, i.e., repeats which are identical in length and located in homologus regions were defined as shared repeats. Under this criteria, there were four repeats shared by all five species. All tested plastomes possessed their own repeats, with the number varying from 13 (*Hepatica henryi*) to 32 (*Pulsatilla chinesis*; Figure 5). These repeats may serve as potential population genetic markers for further studies.

### SSR polymorphisms

We identified 57, 57, 43, 51, and 61 SSRs in *Anemone tomentosa, A. trullifolia, Anemoclema glaucifolium, H. henryi*, and *P. chinensis*, respectively (Table 4). A mononucleotide repeat unit (A/T) was found to be the most abundant, accounting for 65–95% among the five species. This was followed by a dinucleotide repeat unit (AT/AT) with particular numbers of four, six, one, three and two SSRs, respectively. The trinucleotide repeat unit (AAT/ATT) was detected in *Anemone trullifolia, Hepatica henryi*, and *Pulsatilla chinensis*, and only one tetranucleotide repeat (AAGT) was found in *Anemone tomentosa*. Multiple pentanucleotide repeats were detected in *Anemone tomentosa, Hepatica henryi*, and *Pulsatilla chinese*. One hexanucleotide repeat was present in *Pulsatilla chinensis* plastome. The mononucleotide repeat unit C/G was also identified in all five species. Within the five plastomes, SSR loci were mainly located in IGS, followed by locations in CDS and introns. As expected, most SSRs were located in the LSC region, followed by SSC and IR regions.

**Table 4:** Simple sequence repeats (SSRs) for five newly sequenced plastome samples.

**Genomes**	**Repeat units**	**Number**	**Location**			**Region**		
			**Intron**	**IGS**	**CDS**	**LSC**	**SSC**	**IR**
*Anemone tomentosa*	A/T	48	4	39	5	38	6	4
	C/G	1			1	1		
	AT/AT	4		4		4		
	AAGT/ACTT	1		1		1		
	AAAAG/CTTTT	2		2				2
	AATAT/ATATT	1		1		1		
*Anemone trullifolia*	A/T	47	8	31	8	38	5	4
	C/G	2		1	1	2		
	AT/AT	6	1	5		4	2	
	AAT/ATT	2		2				2
*Hepatica henryi*	A/T	33	5	23	5	28	5	
	C/G	1			1	1		
	AT/AT	3	1	2		2	1	
	AAT/ATT	1		1		1		
	AAATT/AATTT	1		1			1	
	AAGAT/ATCTT	1		1			1	
	AATAT/ATATT	1		1			1	
*Pulsatilla chinensis*	A/T	46	7	32	7	36	8	2
	C/G	4	2	2		2		2
	AT/AT	2		1		1		
	AAT/ATT	3		3		1		2
	AAATT/AATTT	1		1		1		
	AAAGT/ACTTT	1		1		1		
	AAGAT/ATCTT	1		1		1		
	AATAT/ATATT	2		2		1	1	
	AATTAT/AATTAT	1		1		1		
*Anemoclema glaucifolium*	A/T	41	5	28	8	27	9	4
	C/G	1		1		1		
	AT/AT	1		1		1		

### Phylogenomic analysis

**Figure 6:**
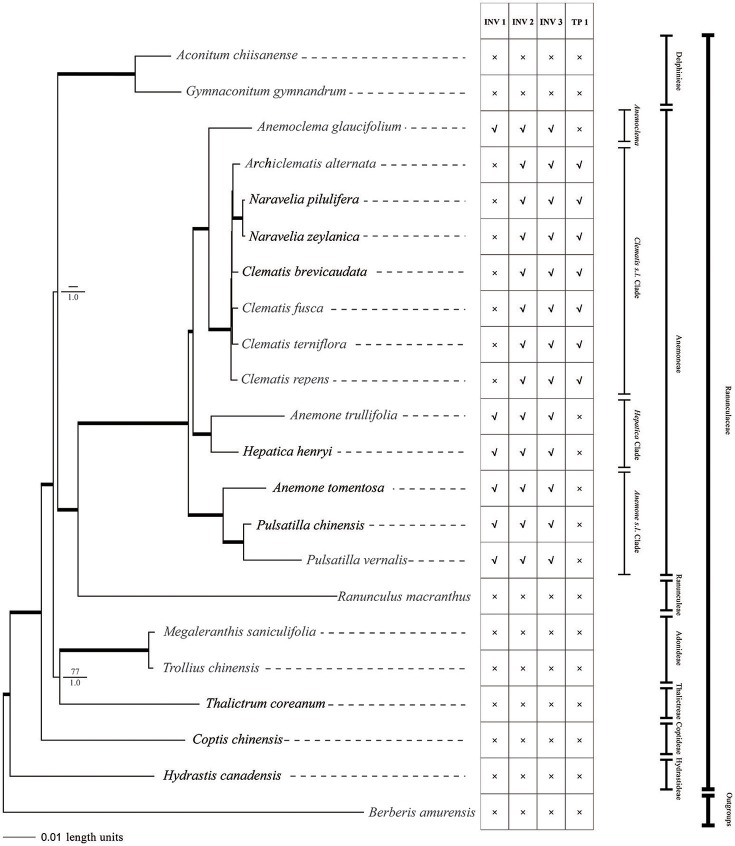
Phylogeny of Ranunculaceae species inferred from complete plastome sequences using MP and Bayesian methods. Bayesian phylograms are shown with MP bootstrap values/PP values for Bayesian analysis at each node. Internal branches which are fully supported by both analyses (with 100 bootstrap values and 1 Bayesian values) were thickened. Plastome Structural variations are also shown. INV, inversion; TP, transposition; ×, absent; √, present.

Phylogenies, reconstructed with each data set and by both methods were consistent with each other and only differed for some nodes' supporting values. Because complete plastome sequence data provided the most robust phylogeny, we used this result (Figure 6). Excluding ambiguous aligments, the complete chloroplast genome data set alignment was 123,519 bp in length (including 16,916 informative characters). Only one parsimony tree with 58,536 steps was searched, along with a consistency index (CI) of 0.75, and a retention index (RI) of 0.75. The Bayesian analysis used partitioned substitution models checked by PartitionFinder. The length of each coding region, intron, intergenic spacer, and models of each partition subset tested by PartitionFinder are provided in the Supplementary Material.

Parsimony analysis of the major Ranunculaceae clades did not resolve the sister relationship of *Aconitum* clade and *Ranunculus* + tribe Anemoneae clade. The *Thalictrum, Trollius*, and *Megaleranthis* clade was also not fully supported by MP analysis (Figure 6). The Bayesian analysis using partitioned models resolved all of the family's major clades. Except for the *Clematis s.l*. clade, all clades were fully supported (PP = 1) by the Bayesian method.

tribe Anemoneae was supported by our phylogenomic analyses and was closely related to *Ranunculus*. There was strong support for grouping *Archiclematis, Clematis*, and *Naravelia* as a sister clade to *Anemoclema*. Samples of *Anemone, Pulsatilla*, and *Hepatica* did not group as a clade but as a paraphyletic grade in tribe Anemoneae. The *Hepatica henryi* + *Anemone trullifolia* (sect. *Omalocarpus*) clade was a sister clade to *Clematis* + *Anemoclema*. *Aneome tomentosa* (sect. *Rivularidium*) + *Pulsatilla* clade was found to be the first diverged clade within the tribe.

## Discussion

### Structual rearrangements of chloroplast genome detected in tribe anemoneae

We found two derived types of chloroplast genomes in tribe Anemoneae compared to other genera within Ranunculaceae, with one type (with two inversions and one transposition regions) in *Clematis s.l*. and the other (with three inversions) in the rest of the tribe's genera (Figures 1, 6). These gene rearrangements clearly bear important phylogenetic information. Two inversions (INV2 and INV3) in *Clematis s.l*. plastome were also present in *Anemoclema, Anenome, Hepatica*, and *Pulsatilla* plastomes. Thus, the presence of INV2 and INV3 could be considered as a synapomorphy of tribe Anemoneae. The largest inversion (INV 1) is present in *Anemoclema, Anemone, Hepatica*, and *Pulsatilla* plastomes, and these samples were paraphyletic to *Clematis s.l*. clade (Figure 6). This suggests that the presence of INV 1 may be a pleisiomorphy within the tribe. In contrast, the only transposition region (TP 1) present in the *Clematis s.l*. clade may represent a synapomorphy for *Clematis, Archiclematis*, and *Naravelia*. The phylogenomic results suggest that the ancestor of tribe Anemoneae may have a plastome sequence similar to that of *Anemone, Anemoclema, Hepatica*, and *Pulsatilla* which carried three inversions in its LSC regions. Two steps of gene rearrangements subsequently occurred in the plastome sequence of the *Clematis s.l*. ancestor. One of these was the loss of INV I, and the other was the addition of TP1.

Inversion and transposition events in the chloroplast genome may be triggered by tRNA activity (Hiratsuka et al., 1989; Walker et al., 2014) or intragenomic recombination at regions with variable G + C content (Fullerton et al., 2001; Smith et al., 2002). In this study, we evaluated the G + C content at the boundaries of each inversion and relocation (Table 3). The regions flanking inversions and relocations had lower G + C contents than non-coding regions of the whole plastome. In contrast, the flanks of all inversion/transposition regions had tRNA genes, as well as higher genetic variation (Figure 4). The tRNA activity, higher genetic variation, and lower G + C content present in flank regions could be the key factors promoting gene rearrangements in chloroplast genomes.

### IR expansion, gene duplications, and other genomic features in tribe anemoneae

Although genome size and overall genomic structure are highly conserved in land plants, IR expansion/contraction is common in plastid genomes and is the main outcome of plastid gemone length variation in angiosperms (Kim and Lee, 2004). Gene duplications in plastid genomes are mainly caused by the expansion of the IR region to single copy regions (Goulding et al., 1996). Expansion events that result in the duplication of single genes, parts of genes, or several genes, have been documented in several plant taxa. This includes a 12-kb expansion in *Nicotiana acuminata* (Goulding et al., 1996), 4-kb expansion in *Jasminum nudiflorum* (Lee et al., 2007), and a remarkable 50-kb expansion in *Pelargonium* (Chumley et al., 2006). In Ranunculaceae, the termini of two genes, *rps19* and *trnH-GUG*, were previously reported to have migrated into adjacent IRs (Park et al., 2015).

We observed an approximately 4.4 kb expansion of the IR toward the LSC region in tribe Anemoneae. This expansion caused duplicate copies of six ribosomal protein genes (*rps8, rpl14, rpl16, rps3, rpl22, rps19*). These are single genes in other genera of the family, such as *Ranunculus, Thalictrum, Megaleranthis, Trollius*, and *Aconitum* (Hoot and Palmer, 1994; Chen et al., 2015; Park et al., 2015), as well as most other angiosperms. For this reason, this IR expansion could also be considered as a synapomorphy of tribe Anemoneae.

Simple sequence repeats (SSRs), also known as microsatellites, are often used as genetic markers for population genetics studies. This is because they provide rich information for population genetics and evolutionary studies (Powell et al., 1995). However, plastid SSRs were rarely used in tribe Anemoneae. We identified 43 to 61 chloroplast SSRs in the five samples we evaluated (Table 4). Our results showed that *Anemone tomentosa, Hepatica henryi*, and *Pulsatilla chinensis* have pentanucleotide repeats in their plastomes, and that the *Pulsatilla chinensis* plastome has hexanucleotide repeats. The rich diversity of chloroplast SSR loci provides opportunities to survey the population genetic structure of those species.

### Phylogenomic inference

All previous phylogenetic studies of Ranunculaceae were based on small numbers of DNA regions (Ro et al., 1997; Wang et al., 2009, 2016; Cossard et al., 2016), and there is a need for improved resolution of Ranunculaceae phylogeny needs to be further improved. In this study, phylogeny inferred from complete chloroplast genomic data was better resolved and more rigorous than previous studies (Figure 6), thereby demonstrating that plastome sequences may provide the ideal data sets for resolving family phylogenies. tribe Anemoneae was supported and tested to be sister to the genus *Ranunculus*, which is in agreement with morphological classifications (Tamura, 1995) and previous molecular phylogenetic studies (Wang et al., 2009, 2016; Cossard et al., 2016). Although the gene order of *Anemoclema* plastome was found to be identical with *Anemone* (Figure 1), this genus has a sister relationship to *Clematis* clade (Figure 6) as previously reported by molecular phylogenetic analyses (Zhang et al., 2015; Jiang et al., 2017a). Unlike results by Hoot et al. (2012), samples of *Anemone, Hepatica*, and *Pulsatilla* did not form a monophyletic group. Thus, our results did not supported classification by Hoot et al. (2012), which included *Hepatica* into *Anemone s.l*.

In this study, *Hetapica henryi*, and *Anemone trullifolia* (sect. *Omalocarpus*) are grouped together, whereas *Pulsatilla chinensis* and *Anemone tomentosa* (sect. *Rivularidum*) are grouped and these two clades were paraphyletic to the *Anemoclema* + *Clematis* clade (Figure 6). These results are somewhat similar to the phylogenetic analyses by Jiang et al. (2017a), but still different with their phylogenetic topology. In Jiang et al. (2017a), *Pulsatilla* + sect. *Rivularidum* clade was sister to *Anemoclema* + *Clematis* clade, and *Hepatica* + sect. *Omalocarpus* clade was outside. In the present study, *Hepatica* + sect. *Omalocarpus* clade was sisiter to *Anemoclema* + *Clematis* clade, whereas *Pulsatilla* + sect. *Rivularidum* clade was outside. Statistical support of sister relationshisp of *Pulsatilla* + sect. *Rivularidum* clade and *Anemoclema* + *Clematis* clade by Jiang et al. (2017a) was not very strong. However, the sister relationship of *Hepatica* + sect. *Omalocarpus* and *Anemoclema* + *Clematis* clade was fully supported by both MP and Bayesian analyses by plastome phylogenomic analyses in this study.

Within tribe Anemoneae, generic relationship within subtribe Clematidinae (sensu Tamura, 1995) was clear. All the small genera like *Archiclematis* and *Naravelia* should be included into *Clematis s.l*. (Miikeda et al., 2006; Xie et al., 2011; Zhang et al., 2015; Liu et al., 2018; Jiang et al., 2017a), and its sister relationship was supported by all the analyses (Zhang et al., 2015; Jiang et al., 2017a; and this study). However, subtribe Anemoninae (sensu Tamura, 1995) showed very complicated evolutionary patterns. Simply grouped together and treated them as *Anemone s.l*. should be reconsidered. According to this study, subtribe Anemoninae should be separated at least three genera (*Anemoclema, Anemone s.l*. including *Pulsatilla* and *Pulsatilloides*, and *Hepatica* including sect. *Omalocarpus*, sect. *Anemonidium*, and sect. *Keiskea*) as suggested by Jiang et al. (2017a), and *Anemoclema* is better to be treated as a member of subtribe Clematidinae.

## Author contributions

HL and JH contributed equally. LX and JC initiated the project. LX, JH, HL, CD, and LP conceived and designed the experiments. HL, JH, and RL performed the experiments. JH and HL analyzed the data. HL, JH, and LX wrote the manuscript.

### Conflict of interest statement

The authors declare that the research was conducted in the absence of any commercial or financial relationships that could be construed as a potential conflict of interest.

## Supplementary material

Figure S1 MAUVE alignment of Ranunculaceae plastomes. Homologous regions are shown in the same color.

Table S1 Detailed informations of gaps between contigs in *de novo* assembly, and primers for Sanger sequencing that bridges the gaps.

Table S2 Results of substitution model and data partition by PartionFinder.

## References

[B1] APG IV (2016). An update of the Angiosperm Phylogeny Group classification for the orders and families of flowering plants: APG IV. Bot. J. Linn. Soc. 181, 1–20. 10.1111/boj.12385

[B2] Brudno M. Do C. B. Cooper G. M. Kim M. F. Davydov E. Green E. D.. (2003a). LAGAN and Multi-LAGAN: efficient tools for large-scale multiple alignment of genomic DNA outline of algorithms. Genome Res. 13, 721–731 10.1101/gr.926603 12654723 PMC430158

[B3] Brudno M. Malde S. Poliakov A. Do C. B. Couronne O. Dubchak I.. (2003b). Glocal alignment: finding rearrangements during alignment. Bioinformatics 19, i54–i62. 10.1093/bioinformatics/btg1005 12855437

[B4] Chen X. Li Q. Li Y. Qian J. Han J. (2015). Chloroplast genome of *Aconitum barbatum* var. puberulum (Ranunculaceae) derived from CCS reads using the PacBio RS platform. Front. Plant Sci. 6:42. 10.3389/fpls.2015.00042 25705213 PMC4319492

[B5] Chumley T. W. Palmer J. D. Mower J. P. Boore J. L. Fourcade H. M. Caile P. J.. (2006). The completed chloroplast genome sequence of *Pelargonium* × *hortorum*: organization and evolution of the largest and most highly rearranged chloroplast genome of land plants. Mol. Biol. Evol. 23, 2175–2190. 10.1093/molbev/msl089 16916942

[B6] Compton J. A. Culham A. Jury S. L. (1998). Reclassification of *Actaea* to include *Cimicifuga* and *Souliea* (Ranunculaceae): phylogeny inferred from morphology, nrDNA ITS, and cpDNA *trnL-F* sequence variation. Taxon 47, 593–634. 10.2307/1223580

[B7] Compton J. A. Hedderson T. A. (1997). A morphometric analysis of the *Cimicifuga foetida* L. complex (Ranunculaceae). Bot. J. Linn. Soc. 123, 1–23. 10.1111/j.1095-8339.1997.tb01402.x

[B8] Cossard G. Sannier J. Sauquet H. Damerval C. de Craene L. R. Jabbour F. (2016). Subfamilial and tribal relationships of Ranunculaceae: evidence from eight molecular markers. Plant Syst. Evol. 302, 419–431. 10.1007/s00606-015-1270-6

[B9] Darling A. E. Mau B. Perna N. T. (2010). progressiveMauve: multiple genome alignment with gene gain, loss and rearrangement. PloS ONE 5:e11147. 10.1371/journal.pone.0011147 20593022 PMC2892488

[B10] Doyle J. J. Doyle J. L. (1987). A rapid DNA isolation procedure for small quantities of fresh leaf tissue. Phytochem. Bull. 19, 11–15.

[B11] Doyle J. J. Doyle J. L. Ballenger J. A. Palmer J. D. (1996). The distribution and phylogenetic significance of a 50-kb chloroplast DNA inversion in the flowering plant family Leguminosae. Mol. Phylogenet. Evol. 5, 429–438. 10.1006/mpev.1996.0038 8728401

[B12] Emadzade K. Gehrke B. Linder H. P. Hörandl E. (2011). The biogeographical history of the cosmopolitan genus *Ranunculus* L.(Ranunculaceae) in the temperate to meridional zones. Mol. Phylogenet. Evol. 58, 4–21. 10.1016/j.ympev.2010.11.002 21078403

[B13] Emadzade K. Lehnebach C. Lockhart P. Hörandl E. (2010). A molecular phylogeny, morphology and classification of genera of *Ranunculeae (Ranunculaceae)*. Taxon 59, 809–828. 10.2307/25677670

[B14] Falck D. Lehtonen S. (2014). Two new names in *Clematis* (Ranunculaceae). Phytotaxa 163, 58 10.11646/phytotaxa.163.1.7

[B15] Frazer K. A. Pachter L. Poliakov A. Rubin E. M. Dubchak I. (2004). VISTA: computational tools for comparative genomics. Nucleic Acids Res. 32(Suppl. 2), W273–W279. 10.1093/nar/gkh458 15215394 PMC441596

[B16] Fullerton S. M. Bernardo Carvalho A. Clark A. G. (2001). Local rates of recombination are positively correlated with G + C content in the human genome. Mol. Biol. Evol. 18, 1139–1142. 10.1093/oxfordjournals.molbev.a003886 11371603

[B17] Gish W. States D. J. (1993). Identification of protein coding regions by database similarity search. Nat. Genet. 3, 266–272. 10.1038/ng0393-266 8485583

[B18] Goulding S. E. Olmstead R. G. Morden C. W. Wolfe K. H. (1996). Ebb and flow of the chloroplast inverted repeat. Mol. Gen. Genet. 252, 195–206. 10.1007/BF02173220 8804393

[B19] Hiratsuka J. Shimada H. Whittier R. Ishibashi T. Sakamoto M. Mori M.. (1989). The complete sequence of the rice (*Oryza sativa*) chloroplast genome: intermolecular recombination between distinct tRNA genes accounts for a major plastid DNA inversion during the evolution of the cereals. Mol. Gen. Genet. 217, 185–194. 10.1007/BF02464880 2770692

[B20] Hoot S. B. Meyer K. M. Manning J. C. (2012). Phylogeny and reclassification of *Anemone* (Ranunculaceae), with an emphasis on austral species. Syst. Bot. 37, 139–152. 10.1600/036364412X616729

[B21] Hoot S. B. Palmer J. D. (1994). Structural rearrangements, including parallel inversions, within the chloroplast genome of *Anemone* and related genera. Mol. Evol. 38, 274–281. 10.1007/BF00176089 8006994

[B22] Hörandl E. Greilhuber J. Klímová K. Paun O. Temsch E. Emadzade K. (2009). Reticulate evolution and taxonomic concepts in the *Ranunculus auricomus* complex (Ranunculaceae): insights from analysis of morphological, karyological and molecular data. Taxon, 58, 1194–1216. 20401184 PMC2855680

[B23] Huang D. I. Cronk Q. C. (2015). Plann: a command-line application for annotating plastome sequences. Appli. Plant Sci. 3:1500026. 10.3732/apps.1500026 26312193 PMC4542940

[B24] Jabbour F. Renner S. S. (2012). A phylogeny of Delphinieae (Ranunculaceae) shows that *Aconitum* is nested within *Delphinium* and that Late Miocene transitions to long life cycles in the Himalayas and Southwest China coincide with bursts in diversification. Mol. Phylogenet. Evol. 62, 928–942. 10.1016/j.ympev.2011.12.005 22182994

[B25] Jansen R. K. Raubeson L. A. Boore J. L. Chumley T. W. Haberle R. C. Wyman S. K.. (2005). Methods for obtaining and analyzing whole chloroplast genome sequences. Methods Enzymol. 395, 348–384. 10.1016/S0076-6879(05)95020-9 15865976

[B26] Jansen R. K. Wojciechowski M. F. Sanniyasi E. Lee S. B. Daniell H. (2008). Complete plastid genome sequence of the chickpea (*Cicer arietinum*) and the phylogenetic distribution of *rps12* and *clpP* intron losses among legumes (Leguminosae). Mol. Phylogenet. Evol. 48, 1204–1217. 10.1016/j.ympev.2008.06.013 18638561 PMC2586962

[B27] Jiang N. Zhou Z. Yang J. B. Yu W. B. (2017b). Complete chloroplast genome of *Anemoclema glaucifolium* (Ranunculaceae), a vulnerable and threatened species endemic to the Hengduan Mountains. Conserv. Genet. Resour. 2017, 1–4. 10.1007/s12686-017-0874-2

[B28] Jiang N. Zhou Z. Yang J. B. Zhang S. D. Guan K. Y. Tan Y. H.. (2017a). Phylogenetic reassessment of tribe Anemoneae (Ranunculaceae): non-monophyly of *Anemone s.l. revealed by plastid datasets*. PloS ONE 12:e0174792. 10.1371/journal.pone.0174792 28362811 PMC5376084

[B29] Johansson J. T. (1999). There large inversions in the chloroplast genomes and one loss of the chloroplast generps16 suggest an early evolutionary split in the genus *Adonis* (Ranunculaceae). Plant Syst. Evol. 218, 133–143. 10.1007/BF01087041

[B30] Katoh K. Kuma K. Toh H. Miyata T. (2005). MAFFT version 5: improvement in accuracy of multiple sequence alignment. Nucleic Acids Res. 33, 511–518. 10.1093/nar/gki198 15661851 PMC548345

[B31] Kearse M. Moir R. Wilson A. Stones-Havas S. Cheung M. Sturrock S.. (2012). Geneious Basic: an integrated and extendable desktop software platform for the organization and analysis of sequence data. Bioinformatics 28, 1647–1649. 10.1093/bioinformatics/bts199 22543367 PMC3371832

[B32] Kim K. J. Lee H. L. (2004). Complete chloroplast genome sequences from Korean ginseng (*Panax schinseng* Nees) and comparative analysis of sequence evolution among 17 vascular plants. DNA Res. 11, 247–261. 10.1093/dnares/11.4.247 15500250

[B33] Kim Y. K. Park C. W. Kim K. J. (2009). Complete chloroplast DNA sequence from a Korean endemic genus, Megaleranthis saniculifolia, and its evolutionary implications. Mol. cells 27, 365–381. 10.1007/s10059-009-0047-6 19326085

[B34] Kumar S. Stecher G. Tamura K. (2016). MEGA7: molecular evolutionary genetics analysis version 7.0 for bigger datasets. Mol. Biol. Evol. 33, 1870–1874. 10.1093/molbev/msw054 27004904 PMC8210823

[B35] Kurtz S. Choudhuri J. V. Ohlebusch E. Schleiermacher C. Stoye J. Giegerich R. (2001). REPuter: the manifold applications of repeat analysis on a genomic scale. Nucleic Acids Res. 29, 4633–4642. 10.1093/nar/29.22.4633 11713313 PMC92531

[B36] Lanfear R. Frandsen P. B. Wright A. M. Senfeld T. Calcott B. (2016). PartitionFinder 2: new methods for selecting partitioned models of evolution for molecular and morphological phylogenetic analyses. Mol. Biol. Evol. 34, 772–773. 10.1093/molbev/msw260 28013191

[B37] Lee H. L. Jansen R. K. Chumley T. W. Kim K. J. (2007). Gene relocations within chloroplast genomes of *Jasminum* and *Menodora* (Oleaceae) are due to multiple, overlapping inversions. Mol. Biol. Evol. 24, 1161–1180. 10.1093/molbev/msm036 17329229

[B38] Lehtonen S. Christenhusz M. J. Falck D. (2016). Sensitive phylogenetics of *Clematis* and its position in Ranunculaceae. Bot. J. Linn. Soc. 182, 825–867. 10.1111/boj.12477

[B39] Li M. Yang B. Chen Q. Zhu W. Ma J. Tian J. (2016). The complete chloroplast genome sequence of *Clematis terniflora* DC.(Ranunculaceae). Mitochondrial DNA Part A 27, 2470–2472. 10.3109/19401736.2015.1033702 25865739

[B40] Librado P. Rozas J. (2009). DnaSP v5: a software for comprehensive analysis of DNA polymorphism data. Bioinformatics 25, 1451–1452. 10.1093/bioinformatics/btp187 19346325

[B41] Lim C. E. Kim G. B. Baek S. Han S. M. Yu H. J. Mun J. H. (2017). The complete chloroplast genome of *Aconitum chiisanense* Nakai (Ranunculaceae). Mitochondrial DNA Part A 28, 75–76. 10.3109/19401736.2015.1110805 26709548

[B42] Liu H. J. Ding C. H. He J. Cheng J. Pei L. Y. Xie L. (2018). Complete chloroplast genomes of *Archiclematis, Naravelia* and *Clematis* (Ranunculaceae), and their phylogenetic implications. Phytotaxa, 343, 214–226. 10.11646/phytotaxa.343.3.2

[B43] Lohse M. Drechsel O. Kahlau S. Bock R. (2013). OrganellarGenomeDRAW-a suite of tools for generating physical maps of plastid and mitochondrial gemones and visualizing expression data sets. Nucleic Acids Res. 41, W575–W581. 10.1093/nar/gkt289 23609545 PMC3692101

[B44] Ma P. F. Zhang Y. X. Zeng C. X. Guo Z. H. Li D. Z. (2014). Chloroplast phylogenomic analyses resolve deep-level relationships of an intractable bamboo tribe Arundinarieae (Poaceae). Syst. Biol. 63, 933–950. 10.1093/sysbio/syu054 25092479

[B45] Mardanov A. V. Ravin N. V. Kuznetsov B. B. Samigullin T. H. Antonov A. S. Kolganova T. V.. (2008). Complete sequence of the duckweed (*Lemna minor*) chloroplast genome: structural organization and phylogenetic relationships to other angiosperms. J. Mol. Evol. 66, 555–564. 10.1007/s00239-008-9091-7 18463914

[B46] Miikeda O. Kita K. Handa T. Yukawa T. (2006). Phylogenetic relationships of *Clematis* (Ranunculaceae) based on chloroplast and nuclear DNA sequences. Bot. J. Linn. Soc. 152, 153–168. 10.1111/j.1095-8339.2006.00551.x

[B47] Moore M. J. Soltis P. S. Bell C. D. Burleigh J. G. Soltis D. E. (2010). Phylogenetic analysis of 83 plastid genes further resolves the early diversification of eudicots. Proc. Natl. Acad. Sci. U.S.A. 107, 4623–4628. 10.1073/pnas.0907801107 20176954 PMC2842043

[B48] Ogihara Y. Terachi T. Sasakuma T. (1988). Intramolecular recombination of chloroplast genome mediated by short direct-repeat sequences in wheat species. Proc. Natl. Acad. Sci. U.S.A. 85, 8573–8577. 10.1073/pnas.85.22.8573 3186748 PMC282501

[B49] Palmer J. D. Nugent J. M. Herbon L. A. (1987). Unusual structure of *Geranium* chloroplast DNA: a triple-sized inverted repeat, extensive gene duplications, multiple inversions, and two repeat families. Proc. Natl. Acad. Sci. U.S.A. 84, 769–773. 10.1073/pnas.84.3.769 16593810 PMC304297

[B50] Park I. Kim W. J. Yang S. Yeo S. M. Li H. Moon B. C. (2017). The complete chloroplast genome sequence of *Aconitum coreanum* and *Aconitum carmichaelii* and comparative analysis with other *Aconitum* species. PloS ONE 12:e0184257. 10.1371/journal.pone.0184257 28863163 PMC5581188

[B51] Park K. T. Park S. (2016). Complete chloroplast genome of *Clematis fusca* var. coreana (Ranunculaceae). Mitochondrial DNA Part A 27, 4056–4058. 10.3109/19401736.2014.1003841 25629487

[B52] Park S. Jansen R. K. Park S. (2015). Complete plastome sequence of *Thalictrum coreanum* (Ranunculaceae) and transfer of the *rpl32* gene to the nucleus in the ancestor of the subfamily Thalictroideae. BMC Plant Biol. 15:40. 10.1186/s12870-015-0432-6 25652741 PMC4329224

[B53] Posada D. Buckley T. R. (2004). Model selection and model averaging in phylogenetics: advantages of Akaike information criterion and Bayesian approaches over likelihood ratio tests. Syst. Biol. 53, 793–808. 10.1080/10635150490522304 15545256

[B54] Powell W. Morgante M. McDevitt R. Vendramin G. G. Rafalski J. A. (1995). Polymorphic simple sequence repeat regions in chloroplast genomes: applications to the population genetics of pines. Proc. Natl. Acad. Sci. U.S.A. 92, 7759–7763. 10.1073/pnas.92.17.7759 7644491 PMC41225

[B55] Raubeson L. A. Peery R. Chumley T. W. Dziubek C. Fourcade H. M.. (2007). Comparative chloroplast genomics: analyses including new sequences from the angiosperms Nuphar advena and Ranunculus macranthus. BMC Genomics 8:174. 10.1186/1471-2164-8-174 17573971 PMC1925096

[B56] Ro K. E. Keener C. S. McPheron B. A. (1997). Molecular phylogenetic study of the Ranunculaceae: utility of the nuclear 26S ribosomal DNA in inferring intrafamilial relationships. Mole. Phylogen. Evol. 8, 117–127. 10.1006/mpev.1997.0413 9299218

[B57] Ronquist F. Huelsenbeck J. P. (2003). MrBayes 3: bayesian phylogenetic inference under mixed models. Bioinformatics 19, 1572–1574. 10.1093/bioinformatics/btg180 12912839

[B58] Shaw J. Lickey E. B. Schilling E. E. Small R. L. (2007). Comparison of whole chloroplast genome sequences to choose noncoding regions for phylogenetic studies in angiosperms: the tortoise and the hare III. Am. J. Bot. 94, 275–288. 10.3732/ajb.94.3.275 21636401

[B59] Smith N. G. Webster M. T. Ellegren H. (2002). Deterministic mutation rate variation in the human genome. Genome Res. 12, 1350–1356. 10.1101/gr.220502 12213772 PMC186654

[B60] Sun M. Li J. Li D. Shi L. (2017). Complete chloroplast genome sequence of the medical fern *Drynaria roosii* and its phylogenetic analysis. Mitochondrial DNA Part B 2, 7–8. 10.1080/23802359.2016.1275835 PMC7800187 33473696

[B61] Swofford D. L. (2003). PAUP^*^: Phylogenetic Analysis Using Parsimony, version 4.0 b10.

[B62] Szczecinska M. Lazarski G. Bilska K. Sawicki J. (2017). The complete plastid genome and nuclear genome markers provide molecular evidence for the hybrid origin of *Pulsatilla* × *hackelii* Pohl. Turk. J. Bot. 41, 329–337. 10.3906/bot-1610-28

[B63] Tamura M. (1993). Ranunculaceae, in The Families and Genera of Vascular Plants II, eds Kubitzki K. Rohwer J. G. Bittrich V (Berlin: Springer), 563–583. 10.1007/978-3-662-02899-5_67

[B64] Tamura M. (1995). *Clematis* L, in Engler's Die Natürlichen Pflanzenfamilien 2nd Edn, ed Heipko P. (Berlin: Duncker & Humblot), 368–387.

[B65] Tangphatsornruang S. Sangsrakru D. Chanprasert J. Uthaipaisanwong P. Yoocha T. Jomchai N.. (2009). The chloroplast genome sequence of mungbean (*Vigna radiata*) determined by high-throughput pyrosequencing: structural organization and phylogenetic relationships. DNA Res. 17, 11–22. 10.1093/dnares/dsp025 20007682 PMC2818187

[B66] Tangphatsornruang S. Uthaipaisanwong P. Sangsrakru D. Chanprasert J. Yoocha T. Jomchai N.. (2011). Characterization of the complete chloroplast genome of *Hevea brasiliensis* reveals genome rearrangement, RNA editing sites and phylogenetic relationships. Gene 475, 104–112. 10.1016/j.gene.2011.01.002 21241787

[B67] Thiel T. Michalek W. Varshney R. K. Graner A. (2003). Exploiting EST databases for the development and characterization of gene-derived SSR-markers in barley (*Hordeum vulgare* L.). Theor. Appl. Genet. 106, 411–422. 10.1007/s00122-002-1031-0 12589540

[B68] Walker J. F. Jansen R. K. Zanis M. J. Emery N. C. (2015). Sources of inversion variation in the small single copy (SSC) region of chloroplast genomes. Amer. J. Bot. 102, 1–2. 10.3732/ajb.1500299 26546126

[B69] Walker J. F. Zanis M. J. Emery N. C. (2014). Comparative analysis of complete chloroplast genome sequence and inversion variation in *Lasthenia burkei* (Madieae, Asteraceae). Amer. J. Bot. 101, 722–729. 10.3732/ajb.1400049 24699541

[B70] Wang W. Dilcher D. L. Sun G. Wang H. S. Chen Z. D. (2016). Accelerated evolution of early angiosperms: evidence from ranunculalean phylogeny by integrating living and fossil data. J. Syst. Evol. 54, 336–341. 10.1111/jse.12090

[B71] Wang W. Hu H. Xiang X. G. Yu S. X. Chen Z. D. (2010). Phylogenetic placements of *Calathodes* and *Megaleranthis* (Ranunculaceae): Evidence from molecular and morphological data. Taxon 59, 1712–1720.

[B72] Wang W. Li R. Q. Chen Z. D. (2005). Systematic position of *Asteropyrum* (Ranunculaceae) inferred from chloroplast and nuclear sequences. Plant Syst. Evol. 255, 41–54. 10.1007/s00606-005-0339-z

[B73] Wang W. Liu Y. Yu S. X. Gao T. G. Chen Z. D. (2013). *Gymnaconitum*, a new genus of Ranunculaceae endemic to the Qinghai-Tibetan Plateau. Taxon 62, 713–722. 10.12705/624.10

[B74] Wang W. Lu A. M. Ren Y. Endress M. E. Chen Z. D. (2009). Phylogeny and classification of Ranunculales: evidence from four molecular loci and morphological data. Perspect. Plant Ecol. 11, 81–110. 10.1016/j.ppees.2009.01.001

[B75] Warren D. L. Geneva A. J. Lanfear R. (2017). RWTY (R We There Yet): An R package for examining convergence of Bayesian phylogenetic analyses. Mol. Biol. Evol. 34, 1016–1020. 10.1093/molbev/msw279 28087773

[B76] Weitemier K. Straub S. C. Cronn R. C. Fishbein M. Schmickl R. McDonnell A.. (2014). Hyb-Seq: combinging target enrichment and genome skimming for plant phylogenomics. Appl. Plant Sci. 2:1400042. 10.3732/apps.1400042 25225629 PMC4162667

[B77] Wolfe K. H. Li W. H. Sharp P. M. (1987). Rates of nucleotide substitution vary greatly among plant mitochondrial, chloroplast, and nuclear DNAs. Proc. Natl. Acad. Sci. U.S.A. 84, 9054–9058. 10.1073/pnas.84.24.9054 3480529 PMC299690

[B78] Wyman S. K. Jansen R. K. Boore J. L. (2004). Automatic annotation of organellar genomes with DOGMA. Bioinformatics 20, 3252–3255. 10.1093/bioinformatics/bth352 15180927

[B79] Xie L. Wen J. Li L. Q. (2011). Phylogenetic analyses of *Clematis* (Ranunculaceae) based on sequences of nuclear ribosomal ITS and three plastid regions. Syst. Bot. 36, 907–921. 10.1600/036364411X604921

[B80] Yan M. Moore M. J. Meng A. Yao X. Wang H. (2017). The first complete plastome sequence of the basal asterid family Styracaceae (Ericales) reveals a large inversion. Plant Syst. Evol. 303, 61–70. 10.1007/s00606-016-1352-0

[B81] Zhang Y. Kong H. H. Yang Q. E. (2015). Phylogenetic relationships and taxonomic status of the monotypic Chinese genus *Anemoclema* (Ranunculaceae). Plant Syst. Evol. 301, 1335–1344. 10.1007/s00606-014-1160-3

