# Supplementary figures and images for: Comparative Analysis of Complete Chloroplast Genomes of Anemoclema, Anemone, Pulsatilla, and Hepatica Revealing Structural Variations Among Genera in Tribe Anemoneae (Ranunculaceae)
Source: Front Plant Sci. 2018 Jul 27;9:1097. doi: 10.3389/fpls.2018.01097 (PMC6073577; doi:10.3389/fpls.2018.01097)

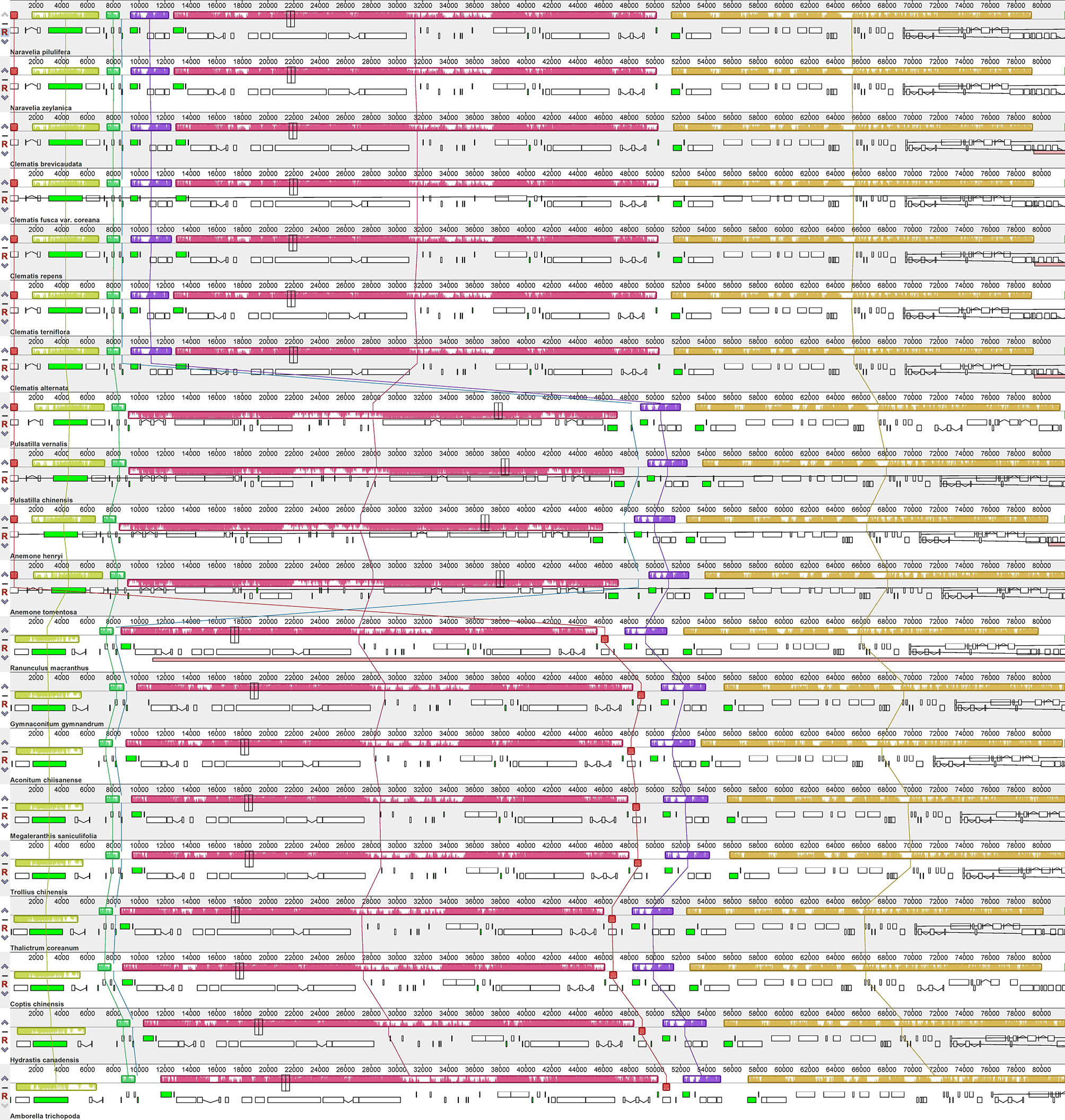

Supplement: Figure S1 — MAUVE alignment of Ranunculaceae plastomes. Homologous regions are shown in the same color. [file Image_1.TIF]
