# Supplementary material for: Comparative Analysis of Complete Chloroplast Genomes of Anemoclema, Anemone, Pulsatilla, and Hepatica Revealing Structural Variations Among Genera in Tribe Anemoneae (Ranunculaceae)
Source: Front Plant Sci. 2018 Jul 27;9:1097. doi: 10.3389/fpls.2018.01097 (PMC6073577; doi:10.3389/fpls.2018.01097)
Supplement: Table S1 — Detailed informations of gaps between contigs in de novo assembly, and primers for Sanger sequencing that bridges the gaps. [file Table_1.DOCX]

Table S1 The designed primers of the gaps for Sanger sequencing.

| **Species** | **Gap length** | |  | **Primers** |
| --- | --- | --- | --- | --- |
| *Hepatica henryi* | 657 bp | | gap1F | AAATAAAACCGAAAGACCCCTTAACT |
|  |  | | gap1R | ACTTTACCTTAAGCGGATACAATCAAT |
| *Anemone tomentosa* | 513 bp | | gap1F | TCTACATTGGATCTCACCTGAATCG |
|  |  | | gap1R | TGCTTGTTGGGTATTTTGGTTTAAC |
|  | 1400 bp | | gap2F | TGGTCGCCTAACACCTAATAATGTA |
|  |  | | gap2R | GCAGAAAAGATTTTCCTGCTTTATGC |
|  | 1600 bp | | gap3F | TCTTTGCACTCGCTTATTTATGGAA |
|  |  | | gap3R | TCGGGGAGCTTTACTTTAAAGTTATTC |
| *Pulsatilla chinensis* | 700 bp | | gap1F | TTGTTTCCGATTCACCAGCTCTTAT |
|  |  | | gap1R | ATCCTTGGAATTAAGTACTTTGGTCAT |
|  | 800 bp | | gap2F | AGTTATGGTTCATTCGCATTAACATTT |
|  |  | | gap2R | TCCGGATTTCGTTCTCTCATTATCA |
|  | 700 bp | | gap3F | TCTTTTCTTGTAGTCGGTACAGTCA |
|  |  | | gap3R | GCCAAAAACTCGTTAAGCTCATTTT |
|  | 800 bp | | gap4F | TACAACGCGATAAAGTCTCTTCTGA |
|  |  | | gap4R | AAACTCTACCAATGGTATGGACGAA |
|  | 700 bp | | gap5F | TTCACCGATCCATCAATCATGAGAT |
|  |  | | gap5R | CGAAATCCTATTGTCAAATCCGTGT |
|  | 700 bp | | gap6F | CGGGTCTCATTTTAAGATTTGTCGA |
|  |  | | gap6R | TTATATGTAAGGGCTCCTCGCAATT |
|  | 700 bp | | gap7F | TTCTTGTTGATCGATGCAATTAGGG |
|  |  | gap7R | | CACCACAGAAATTCGCATAAAATGG |
| *Anemoclema glaucifolium* | 1000 bp | gap1F | | GATCAGGAATTAGCGGATCCACTC |
|  |  | gap1R | | TCGGTTACATGTTTCATATGATCTCC |
|  | 1200 bp | gap2F | | GTGGCCCGAAAATAATTAGGTTAGA |
|  |  | gap2R | | ACCGCAATGTATCAAATCCAATACA |
