# Supplementary material for: Comparative Analysis of Complete Chloroplast Genomes of Anemoclema, Anemone, Pulsatilla, and Hepatica Revealing Structural Variations Among Genera in Tribe Anemoneae (Ranunculaceae)
Source: Front Plant Sci. 2018 Jul 27;9:1097. doi: 10.3389/fpls.2018.01097 (PMC6073577; doi:10.3389/fpls.2018.01097)
Supplement: Table S2 — Results of substitution model and data partition by PartionFinder. [file Table_2.DOCX]

Table S2

| Protein coding region | accD (1440) atpA (1524) atpB (1497) atpE (402) atpF (555) atpH (246) atpI (744) ccsA (966) cemA (690) clpP (606) infA (165) matK (1527) ndhA (1092) ndhB (1533) ndhC (363) ndhD (1479) ndhE (306) ndhF (2211) ndhG (534) ndhH (1182) ndhI (543) ndhJ (477) ndhK (678) petA (969) petB (648) petD (504) petG (114) petL (96) petN (90) psaA (2253) psaB (2205) psaC (246) psaI (111) psaJ (135) psbA (1062) psbB (1527) psbC (1422) psbD (1062) psbE (252) psbF (120) psbH (222) psbI (111) psbJ (123) psbK (186) psbL (117) psbM (105) psbN (132) psbT (102) psbZ (189) rbcL (1428) rpl14 (369) rpl16 (408) rpl2 (819) rpl20 (354) rpl22 (540) rpl23 (282) rpl32 (143) rpl33 (201) rpl36 (114) rpoA (991) rpoB (3213) rpoC1 (2043) rpoC2 (4137) rps11 (417) rps12 (372) rps14 (303) rps15 (273) rps16 (260) rps18 (306) rps19 (279) rps2 (711) rps3 (657) rps4 (606) rps7 (468) rps8 (399) ycf1 (5478) ycf2 (6807) ycf3 (507) ycf4 (555) |
| --- | --- |
| Transfer RNA coding region | trnA-UGC (73) trnC-GCA (71) trnD-GUC (74) trnE-UUC (73) trnF-GAA (73) trnfM-CAU (74) trnG-GCC (71) trnG-UCC (71) trnH-GUG (74) trnI-CAU (74) trnI-GAU (72) trnK-UUU (72) trnL-CAA (81) trnL-UAA (85) trnL-UAG (80) trnM-CAU (73) trnN-GUU (72) trnP-UGG (74) trnQ-UUG (72) trnR-ACG (74) trnR-UCU (72) trnS-GCU (88) trnS-GGA (87) trnS-UGA (93) trnT-GGU (72) trnT-UGU (73) trnV-GAC (72) trnV-UAC (73) trnW-CCA (74) trnY-GUA (84) |
| Ribonucleic RNA coding region | rrn16 (1491) rrn23 (2809) rrn4.5 (103) rrn5 (121) |
| Nocoding region | intergenic region (32771) gene intron (15650) |
| PartitionFinder Subset | Subset1 = 86663-87202\3 7375-8064\3 1-1440\3;  Subset2 = 82864-82993\3 2-1440\3 99419-99691\3;  Subset3 = 3-1440\3;  Subset4 = 1441-2964\3 2965-4461\3;  Subset5 = 1442-2964\3 122459-122964\3 87829-87942\3;  Subset6 = 94192-98326\3 85084-85489\3 4866-5418\3 86311-86662\3 122967-123519\3 2967-4461\3 1443-2964\3;  Subset7 = 2966-4461\3;  Subset8 = 102979-103377\3 100537-101247\3 63251-63556\3 100258-100536\3 4462-4863\3 4864-5418\3;  Subset9 = 68028-68503\3 92148-94189\3 88935-92146\3 66303-67483\3 4463-4863\3;  Subset10 = 8067-8670\3 7377-8064\3 4464-4863\3 87945-88933\3;  Subset11 = 85492-86308\3 4865-5418\3 101249-101904\3 87944-88933\3;  Subset12 = 109239-109310 108266-108339 107975-108045 87830-87942\3 5419-5664\3;  Subset13 = 79143-80563\3 82332-82453\3 71514-71602\3 5420-5664\3 76419-76552\3;  Subset14 = 99118-99418\3 88936-92146\3 5421-5664\3 92149-94189\3;  Subset15 = 108120-108192 68027-68503\3 5665-6408\3 92147-94189\3 99953-100257\3 101905-102510\3;  Subset16 = 82758-82861\3 5666-6408\3 82641-82756\3 81879-81997\3 83097-83284\3 82995-83095\3 70800-71302\3 61410-61771\3 82221-82330\3 71304-71416\3 70152-70798\3 59877-61408\3;  Subset17 = 99694-99951\3 101250-101904\3 101907-102510\3 5667-6408\3;  Subset18 = 6409-7374\3 63557-65767\3;  Subset19 = 6410-7374\3;  Subset20 = 87831-87942\3 6411-7374\3;  Subset21 = 7376-8064\3;  Subset22 = 8065-8670\3;  Subset23 = 8066-8670\3 57258-58783\3 83098-83284\3 82759-82861\3 71419-71512\3;  Subset24 = 8671-41441;  Subset25 = 41442-41606\3;  Subset26 = 41443-41606\3;  Subset27 = 41444-41606\3;  Subset28 = 41607-57256;  Subset29 = 57257-58783\3 87486-87627\3;  Subset30 = 63253-63556\3 57259-58783\3;  Subset31 = 71418-71512\3 58784-59875\3 65768-66301\3 99693-99951\3 82642-82756\3 81880-81997\3;  Subset32 = 82455-82639\3 63252-63556\3 65769-66301\3 58785-59875\3;  Subset33 = 58786-59875\3 66304-67483\3;  Subset34 = 81626-81877\3 108556-108629 82994-83095\3 109385-109456 102512-102978\3 59876-61408\3;  Subset35 = 69183-70150\3 59878-61408\3;  Subset36 = 98328-98743\3 102980-103377\3 61409-61771\3;  Subset37 = 82456-82639\3 68506-69181\3 68029-68503\3 61411-61771\3 83287-84712\3 73858-76060\3 79144-80563\3 80566-81625\3;  Subset38 = 82331-82453\3 76418-76552\3 82454-82639\3 61772-63250\3;  Subset39 = 61773-63250\3 76308-76417\3;  Subset40 = 98329-98743\3 61774-63250\3 82000-82219\3;  Subset41 = 63558-65767\3 76307-76417\3;  Subset42 = 63559-65767\3;  Subset43 = 99421-99691\3 65770-66301\3 67486-68026\3;  Subset44 = 84713-85081\3 69182-70150\3 88934-92146\3 66302-67483\3 85082-85489\3;  Subset45 = 87943-88933\3 99692-99951\3 101248-101904\3 98327-98743\3 94190-98326\3 67484-68026\3 122965-123519\3;  Subset46 = 100259-100536\3 67485-68026\3 94191-98326\3;  Subset47 = 68505-69181\3 109632-109724 68504-69181\3 100538-101247\3 101906-102510\3 99116-99418\3;  Subset48 = 77617-79141\3 70801-71302\3 69184-70150\3;  Subset49 = 76553-77614\3 77615-79141\3 79142-80563\3 80564-81625\3 71603-73855\3 70151-70798\3 76061-76306\3;  Subset50 = 70153-70798\3;  Subset51 = 70799-71302\3 108482-108555;  Subset52 = 73856-76060\3 109797-109869 71303-71416\3 82862-82993\3 110089-110172 107902-107974 108046-108119 98745-99115\3 109093-109164 109020-109092 109725-109796 110015-110088;  Subset53 = 71305-71416\3 87630-87828\3 100539-101247\3 82333-82453\3 81628-81877\3;  Subset54 = 99952-100257\3 82757-82861\3 87628-87828\3 71417-71512\3;  Subset55 = 83096-83284\3 109457-109544 71513-71602\3 82220-82330\3 81878-81997\3 122458-122964\3 109942-110014 109870-109941;  Subset56 = 87205-87484\3 98746-99115\3 102513-102978\3 71515-71602\3;  Subset57 = 73857-76060\3 80565-81625\3 76554-77614\3 71604-73855\3 82863-82993\3 82640-82756\3 84714-85081\3;  Subset58 = 71605-73855\3;  Subset59 = 83285-84712\3 85083-85489\3 76062-76306\3;  Subset60 = 76063-76306\3 76420-76552\3;  Subset61 = 82222-82330\3 100260-100536\3 122460-122964\3 76309-76417\3;  Subset62 = 76555-77614\3;  Subset63 = 109545-109631 77616-79141\3 109165-109238 81627-81877\3;  Subset64 = 81998-82219\3 87629-87828\3;  Subset65 = 81999-82219\3;  Subset66 = 82996-83095\3;  Subset67 = 83286-84712\3;  Subset68 = 84715-85081\3;  Subset69 = 85490-86308\3 99117-99418\3 107678-107780 102511-102978\3 87203-87484\3 98744-99115\3;  Subset70 = 85491-86308\3 108940-109019 87204-87484\3 108774-108854 108702-108773 108193-108265 107781-107901 109311-109384 108630-108701;  Subset71 = 86309-86662\3;  Subset72 = 86310-86662\3;  Subset73 = 86664-87202\3 99420-99691\3;  Subset74 = 86665-87202\3 102981-103377\3;  Subset75 = 87485-87627\3;  Subset76 = 87487-87627\3;  Subset77 = 99954-100257\3;  Subset78 = 104869-107677 103378-104868;  Subset79 = 108340-108410;  Subset80 = 108411-108481;  Subset81 = 108855-108939;  Subset82 = 110173-115650\3 110175-115650\3;  Subset83 = 110174-115650\3;  Subset84 = 115651-122457\3;  Subset85 = 115652-122457\3;  Subset86 = 115653-122457\3 122966-123519\3; |
|  | Subset1=GTR+G Subset2=GTR+G Subset3=GTR+G  Subset4=GTR+I+G Subset5=GTR+I+G Subset6=GTR+G  Subset7=GTR+G Subset8=GTR+G Subset9=GTR+I+G  Subset10=GTR+G Subset11=GTR+G Subset12=GTR  Subset13=GTR Subset14=GTR+G Subset15=GTR+G  Subset16=GTR+I+G Subset17=GTR+G Subset18=GTR+I+G  Subset19=GTR+I+G Subset20=GTR+G Subset21=GTR+G  Subset22=GTR+G Subset23=GTR+G Subset24=GTR+I+G  Subset25=GTR Subset26=GTR Subset27=GTR  Subset28=GTR+I+G Subset29=GTR+G Subset30=GTR+G  Subset31=GTR+G Subset32=GTR+G Subset33=GTR+G  Subset34=GTR+G Subset35=GTR+G Subset36=GTR+G  Subset37=GTR+G Subset38=GTR+I+G Subset39=GTR+I+G  Subset40=GTR+G Subset41=GTR+I+G Subset42=GTR+G  Subset43=GTR+G Subset44=GTR+I+G Subset45=GTR+G  Subset46=GTR+I+G Subset47=GTR+G Subset48=GTR+G  Subset49=GTR+I+G Subset50=GTR+G Subset51=GTR+I+G  Subset52=GTR+I+G Subset53=GTR+G Subset54=GTR  Subset55=GTR Subset56=GTR Subset57=GTR+I+G  Subset58=GTR+G Subset59=GTR+I+G Subset60=GTR+G  Subset61=GTR+G Subset62=GTR+G Subset63=GTR+I+G  Subset64=GTR Subset65=GTR+I+G Subset66=GTR+G  Subset67=GTR+I+G Subset68=GTR Subset69=GTR+I+G  Subset70=GTR+I+G Subset71=GTR+G Subset72=GTR+G  Subset73=GTR+G Subset74=GTR+G Subset75=GTR+I+G  Subset76=GTR+G Subset77=GTR Subset78=GTR+I+G  Subset79=GTR+I+G Subset80=GTR+G Subset81=GTR+I+G  Subset82=GTR+G Subset83=GTR+G Subset84=GTR+G  Subset85=GTR+G Subset86=GTR+G |
